# The Role of Genetics in Human Oral Health: A Systematic–Narrative Review

**DOI:** 10.3390/dj13030133

**Published:** 2025-03-16

**Authors:** Anita Joy-Thomas, Zarna Lalwani, Leticia Guajardo, John Valenza, Walid D. Fakhouri

**Affiliations:** 1 Department of Diagnostic and Biomedical Sciences, Center for Craniofacial Research, School of Dentistry, University of Texas Health Science Center at Houston, Houston, TX 77054, USA; zarna.p.lalwani@uth.tmc.edu; 2 Department of Endodontics, School of Dentistry, University of Texas Health Science Center at Houston, Houston, TX 77054, USA; leticia.guajardo@uth.tmc.edu; 3 Department of General Practice and Dental Public Health, School of Dentistry, University of Texas Health Science Center at Houston, Houston, TX 77054, USA; john.a.valenza@uth.tmc.edu; 4 Department of Pediatrics, McGovern Medical School, University of Texas Health Science Center at Houston, Houston, TX 77054, USA

**Keywords:** inheritance pattern, oral conditions, dental diseases, genetic contribution, DNA mutations, environmental risk factors

## Abstract

**Background/Objectives**: An individual’s genetic makeup influences their organ development, orofacial structures, and overall health. Though many studies have been conducted to determine the inheritance of oral diseases and conditions, there is a lack of comprehensive research classifying these disorders based on the genetic and environmental etiology. **Methods**: This systematic review aimed to analyze the existing body of literature using the PubMed and Cochrane databases and answer the following question: “What evidence exists supporting the role of genetic factors in oral conditions?” This systematic–narrative review methodically categorizes oral diseases and conditions based on their genetic or environmental linkages. Each classification is rigorously supported by the peer-reviewed articles and evidence strength, affirming the sufficient validity of the identified associations. **Results**: This study provides an overview of how genetics can influence oral health, from predisposition to susceptibility to various oral diseases, and the impact of genetic alterations on dental and oral conditions. Additionally, this study discusses the importance of understanding the interplay between genetic and environmental factors to improve oral health outcomes. An enhanced understanding of the impact of genetics on oral health will provide a better understanding of the implications of inherited or de novo genetic mutations and their potential interactions with environmental factors. **Conclusions**: The data collection and analysis indicate 25 oral conditions with strong genetic components and 2 with moderate genetic contributions (fibrous dysplasia and impacted teeth), while 14 oral conditions seem to have weak genetic contributions. Treatment planning that includes genetic testing and counseling as an approach of precision oral healthcare is encouraged to develop appropriate preventative and timely treatment plans to provide the effective management of patients’ symptoms.

## 1. Introduction

Genetics plays a crucial role in oral health in numerous ways. Several oral health conditions have been linked to genetic mutations and chromosomal abnormalities. The most common oral disease, dental caries, is known to be influenced by a genetic makeup that may increase an individual’s susceptibility by impacting the structure and compromising the strength of the hard tissue of their teeth [1,2]. The risk of developing periodontal diseases like gingivitis or periodontitis may be increased by genetic variations that affect the body’s immune response to oral bacteria [3,4,5]. Often, variables such as food, oral hygiene, and systemic diseases could be thought to have a larger impact on the etiology of the most common oral diseases if the supporting evidence for the role of genetic factors in oral conditions has not been systematically analyzed. Today, research has shown that some genetic mutations, when present, can increase the risk of developing oral cancers [6], while other genetic factors can influence the size, shape, and position of teeth, impacting esthetics, function, and overall oral health. Genetic influences can also affect the development and function of salivary glands, including the amount of saliva produced and the quality of the saliva, both of which can impact oral health. Genetic impacts can even be manifested at birth with the development of craniofacial anomalies, such as cleft lip with/or without cleft palate, which are caused by a combination of genetic and environmental factors [7].

It is essential to understand that while genetics determines the predisposition or risk of contracting a disease, it does not provide certainty that the individual will eventually develop the disease. Most oral diseases have multifactorial causes, including genetics, epigenetics, and environmental factors, that are interlinked. While genetics can contribute to oral health conditions, environmental and lifestyle factors, such as diet, oral hygiene habits, and the use of substances such as tobacco, alcohol, and recreational drugs, also play a significant role and can influence cellular function. Human DNA or genes could be considered analogous to a computer motherboard, which is the main component that controls all operations of a computing device. However, the intensity of the electricity, exposure to heat and cold, the hard disk space, and viral infections are external factors that can influence the “health” of the computer—i.e., its performance and efficiency. Similarly, an individual’s genetics is usually responsible for host factors such as salivary composition, immune responses, the structure of the enamel, dentin, and cementum (hard tissues of a tooth), as well as the sizes and shapes of the jaw, throat, teeth, and tongue. Additionally, environmental influences, such as fluoride exposure, oral hygiene habits, drug exposure, occupational exposure, oxygen levels, and radiation therapy, can influence optimal oral function and health. As such, disease risk and severity may be exacerbated or mitigated by the interactions of intrinsic/genetic and extrinsic/environmental factors. The goal of this study is to systematically review the literature to methodically categorize oral diseases and conditions based on their genetic or environmental linkages based on the number of supported peer-reviewed articles to affirm the genetic contribution to oral conditions.

## 2. Materials and Methods

### 2.1. Standardized Criteria and PRISMA Flowchart

This systematic review followed the Cochrane Collaboration guidelines and the Preferred Reporting Items for Systematic Reviews and Meta-Analysis (PRISMA) criteria. The authors adhered to the current Preferred Reporting Items for Systematic Reviews and Meta-Analysis (PRISMA) criteria, as well as to the recently published models of systematic review to ensure the standardization of the data inclusion/exclusion criteria for studies investigating the cause or contribution of genetic factors in oral conditions.

### 2.2. Search Strategy

An electronic search was conducted using the NCBI and Cochrane databases from 1960 up to May 2024 to ensure a comprehensive analysis of all peer-reviewed articles pertaining to genetic contribution or genetic causes and various oral conditions. However, no articles were retrieved from the 1960–1970 decade in our data analysis. The search encompassed randomized controlled trials (RCTs), pilot studies, clinical trials, case reports, and review articles.

The keywords used in the literature search were “genetics”, or “genome” or “DNA variation” or “DNA mutation” and “Dentinogenesis imperfecta” or “Dental caries” or “Gingivitis” or “Dental Attrition”, or the remaining of the oral conditions, for the NCBI database. Similarly, the keywords used for Cochrane were “genetics”, or “genome” or “DNA variation” or “DNA mutation” AND “Dentinogenesis imperfecta” or “Dental caries” or “Gingivitis” or “Dental Attrition”, or the remaining of the oral conditions, for the article title and abstract. We conducted the search using these keywords for each oral condition separately. Relevant abstracts were downloaded from the NCBI and Cochrane databases. The abstracts of the retrieved articles were assessed to determine whether details related to the indication genetic factors or genetic mutations as the etiology of an oral condition or significant association between DNA mutations and an oral condition were included.

### 2.3. Inclusion Criteria

Articles meeting the following criteria were included: (1) published in English, (2) randomized controlled trails (RCTs), pilot studies, clinical studies, or animal studies, and (3) focused on genetic factors or DNA mutations leading or contributing to oral and dental disorders.

### 2.4. Exclusion Criteria

Articles meeting the following criteria were excluded: (1) reviews, (2) articles pertaining to epigenetics or environmental factors, (3) case reports not focused on genetic testing and genotyping, (4) duplicates, (5) questions not focused, and (6) published in a language other than English.

### 2.5. Data Collection and Analysis Process

Two calibrated curators independently reviewed all titles and abstracts for the inclusion and exclusion criteria, followed by a second review in case of any disagreement. The entire retrieved article was evaluated in case of any disagreements between the two curators regarding inclusion or exclusion. A third previously calibrated reviewer reviewed the articles that raised disagreement among the two curators. An evidence strength for the included articles was developed for the data analysis out of the total retrieved articles from the literature search as follows:

Evidence strength 0 for < 10 articles’ focus on genetic contribution or causes;
Evidence strength 1 for ≥ 10 articles’ focus on genetic contribution or causes;
Evidence strength 2 for ≥ 20 articles’ focus on genetic contribution or causes;
Evidence strength 3 for ≥ 30 articles’ focus on genetic contribution or causes;
Evidence strength 4 for ≥ 40 articles’ focus on genetic contribution or causes;
Evidence strength 5 for ≥ 50 articles’ focus on genetic contribution or causes.

## 3. Results

**Figure 1:**
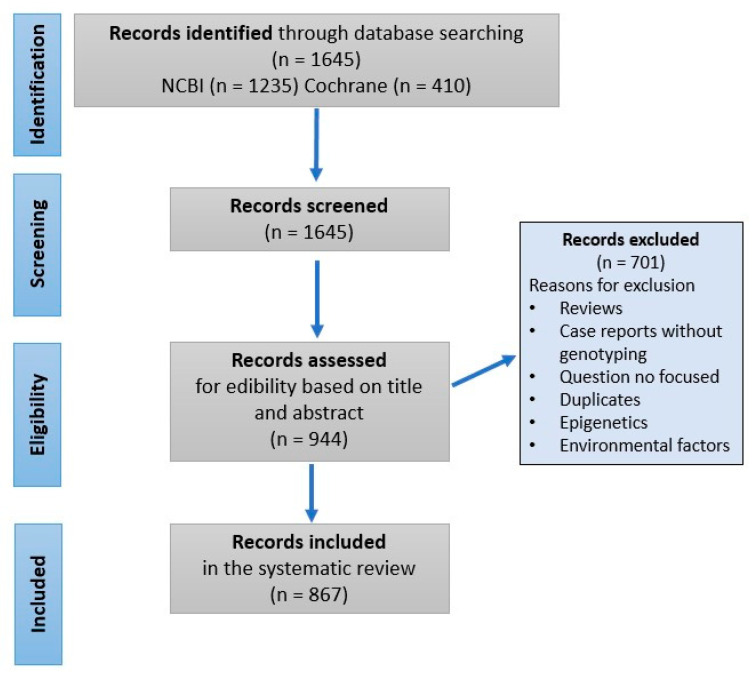
A flowchart of the systematic review representing the literature search using the NCBI and Cochrane databases, literature screening, inclusion and exclusion criteria, and the selection of the retrieved articles in this study.

Initially, 1645 articles were included after completing electronic searches of the NCBI and Cochrane databases. Following screening, we excluded 701 articles based on the exclusion criteria mentioned above. The final systematic review included 860 and 7 publications from the NCBI and Cochrane databases, respectively (Figure 1). A review of these publications resulted in categorizing orofacial disorders into those that have strong, moderate, or weak genetic influence (Table 1). We describe briefly below some of the oral and dental anomalies caused primarily by genetic alterations.

### 3.1. Orofacial Disorders with Strong to Moderate Genetic Components

Oral disorders and diseases that occur at birth or in young children have a vital genetic component. These conditions may be attributed to a single gene disruption or chromosomal abnormality. Environmental factors can influence the incidence and severity of these conditions (Table 1). The limitations of our data analysis are the potential biases in genetic studies due to population diversity and/or environmental interplay.

#### 3.1.1. Tooth Agenesis

Hypodontia

Hypodontia is a condition in which a person has fewer teeth than normal, either because some teeth are missing or fail to develop properly (usually < 6 missing teeth). It is a relatively common condition that affects 5% (95% CI: 4.1–5.9) of the population in North America [8]. In the permanent dentition, teeth that typically exhibit tooth agenesis are third molars (>20%), followed by mandibular second premolars (4.2%), maxillary lateral incisors (2.3%), and maxillary second premolars (2.2%) [9]. Unilateral agenesis is more frequent than bilateral except for the maxillary lateral incisors [10]. Hypodontia can occur as a nonsyndromic, isolated condition or as part of a congenital syndrome. It has been linked to mutations in several key genes, including *PAX9*, *MSX1*, and *AXIN2* [11,12,13,14,15,16,17,18,19]. *PAX9* mutations are particularly significant as they disrupt tooth development through interference with epithelial–mesenchymal interactions crucial for tooth germ and enamel organ formation [12]. *MSX1* is involved in the early stages of dental development, and its mutations can lead to a failure in the development of certain teeth [19]. *AXIN2*, a gene that plays a role in the Wnt signaling pathway, affects the differentiation of dental mesenchymal cells [20]. The etiology of this condition is multifactorial, with strong genetic components and contributions. Twin studies demonstrated that the loss of lateral incisors and premolars is inherited in an autosomal-dominant fashion with variable expressivity and incomplete penetrance [10].

Oligodontia

**Figure 2:**
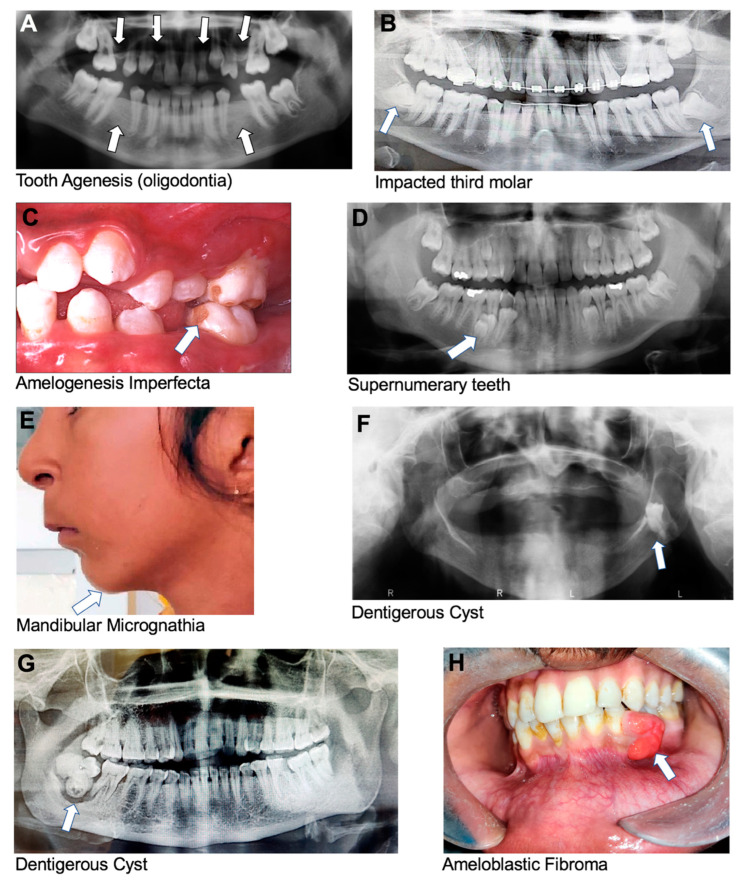
Oral conditions with strong genetic components as per our data analysis of retrieved articles. (**A**) Panoramic radiograph image of individual with oligodontia showing absence of six permanent teeth (excluding third molars). The following teeth are missing: 12, 15, 22, 25, 35, and 45, using the FDI numbering system (courtesy of Dr. Ariadne Letra). (**B**) Panoramic radiograph image of individual with impacted third molars. (**C**) Amelogenesis imperfecta showing enamel pitting with exposed dentin (courtesy of Dr. Cleverick (C.D.) Johnson). (**D**) Supernumerary teeth. (**E**) Mandibular micrognathia showing short lower jaw. (**F**,**G**) Dentigerous cyst (Image G is courtesy of Dr. Ritu Tiwari). (**H**) Ameloblastic fibroma at the lower left quadrant.

Oligodontia is a rare genetic condition that affects the development of teeth during childhood, characterized by six or more missing permanent teeth (Figure 2A), and is similar to hypodontia. Oligodontia can be caused by mutations in various genes involved in tooth development, like *AXIN2*, *MSX1*, and *PAX9* [11,12,13,14,16,20,21,22,23]. *MSX1* mutations impair the initial stages of dental development, while *PAX9* mutations affect epithelial–mesenchymal interactions necessary for early tooth formation [12,19]. The condition can also be associated with other congenital syndromes, such as ectodermal dysplasia, Down syndrome, or familial cleft lip and palate, which are inherited in an autosomal-dominant pattern [20,21,22,23].

Anodontia

Anodontia is a rare genetic condition characterized by the complete absence of teeth, with individuals not developing primary or permanent teeth. Manifestations include a lack of alveolar ridge development, which reduces the lower face’s vertical dimension and causes the lips’ vermilion border to vanish, giving an elderly look [24,25].

Tooth agenesis is typically caused by genetic mutations in *MSX1*, *PAX9*, and *AXIN2* that affect tooth development [26,27]. In some cases, it can be associated with other syndromic disorders, such as derivatives of ectodermal dysplasia, Van der Woude syndrome, Rieger’s syndrome, and Witkop’s tooth and nail syndrome [28,29,30,31].

#### 3.1.2. Tooth Shape Alterations

Taurodontism

Taurodontism is a condition that affects the shape and size of teeth, particularly molars. It is characterized by the elongation of the pulp chamber and the apical displacement of the furcation, resulting in a rectangular or cylindrical tooth shape. The roots of taurodont teeth are often shorter and thicker than those of normal teeth [32]. Taurodontism can be a hereditary condition or associated with certain genetic syndromes, such as Klinefelter syndrome (extra copy of X chromosome) and Down syndrome (trisomy 21), which are inherited in a dominant pattern [32,33,34]. It can also occur due to certain environmental factors, such as radiation exposure. Individuals with taurodontism may not experience any symptoms, but it can make dental procedures more challenging due to the altered shape and size of the teeth [34].

Peg-shaped Laterals

Peg laterals, also known as peg-shaped or small lateral incisors, are a dental condition where the lateral incisors are smaller than usual and have a conical shape, instead of the usual rectangular shape, and peg laterals are more pointed and tapered [35]. This condition is typically a result of genetics and can affect the lateral incisors unilaterally or bilaterally [36].

#### 3.1.3. Tooth Size Abnormalities

Microdontia

Microdontia is characterized by the presence of abnormally small teeth, which are smaller than the normal size for a person’s age and sex. Microdontia can be classified as localized/isolated, true generalized, or relatively generalized [37], and it most commonly affects the upper lateral incisors. Microdontia is multifactorial with a strong genetic contribution and environmental modifications during tooth development, or it can be caused due to certain medical conditions, such as pituitary dwarfism (no definitive single genetic cause) [38] or Down syndrome [9,39,40].

Macrodontia

Macrodontia is characterized by abnormally large teeth, which are larger than the normal size for a person’s age and sex. Macrodontia can be classified as localized/isolated, true generalized, or relatively generalized [32,33]. It is most commonly seen in molars and premolars. Macrodontia can be caused by genetic and environmental factors during tooth development. It is associated with certain syndromes and medical conditions, including otodental syndrome, insulin-resistant diabetes, pituitary gigantism, and hemifacial hyperplasia [9,41,42,43].

#### 3.1.4. Tooth Eruption Discrepancies

Impacted or Embedded Teeth

Impacted teeth are teeth that cannot emerge fully into the mouth due to a lack of space, an obstruction in their eruption path, and/or a misalignment resulting in a lack of an eruptive path; all these factors are influenced by genetics (Figure 2B). This can occur with any tooth in the mouth, but it most commonly affects the third molars, or wisdom teeth, and maxillary canines [44,45].

#### 3.1.5. Tooth Structure Abnormalities

Amelogenesis Imperfecta

Amelogenesis imperfecta (AI) is a genetic disorder that affects the development of tooth enamel, which is the hard, protective, outer layer of teeth. This condition can affect both primary and permanent teeth, and it can result in teeth that are discolored, weak, and prone to fracture [46]. There are several types of AI, each with its own specific pattern of inheritance and symptoms. The disorder can be inherited in an autosomal-dominant, autosomal-recessive, or X-linked manner, depending on the specific genetic mutation involved [28]. Mutations in the genes *AMELX*, *ENAM*, *MMP20*, *KLK4*, and *DLX3* are reported to be associated with the development of AI [9,28,46,47]. Clinically, teeth affected by AI may be yellow or brown, have rough or pitted surfaces, or be abnormally small or misshapen (Figure 2C). Teeth affected by this condition may also be sensitive to temperature and pressure.

Dentinogenesis Imperfecta

Dentinogenesis imperfecta (DI) is a genetic disorder that affects the development of dentin that forms the bulk of the tooth structure beneath the enamel [28]. The prevalence of DI is 1 in 8000 people [48]. This condition can affect both primary and permanent teeth, resulting in teeth that have brown to blue discoloration, are weak, and are prone to attrition and breakage [49]. There are three types of DI, and the specific symptoms and severity of the condition can vary widely depending on the type. Type I is characterized by abnormal dentin with concurrent osteogenesis imperfecta, when the primary teeth are more severely affected. Osteogenesis imperfecta is usually associated with mutations in the genes encoding collagen type 1, *COL1A1*, and *COL1A2* [28,48,50]. In type II of DI, patients have dentin abnormalities, but without bone disorders. Mutations in the gene *DSPP* have been associated with the development of DI type II [28,48]. Type III of DI is a rare variant found in the tri-racial southern Maryland population known as the “Brandywine isolate”. Its clinical characteristics vary and are similar to those observed in DI-I and -II. The primary teeth show multiple pulp exposures and radiographically show a “shell”-like appearance [48,51].

In some cases, teeth affected by DI may be gray or brown in color and have a bulbous shape and a translucent appearance. Like AI, the mutations that cause DI affect the quality and quantity of the dentin produced, leading to the characteristic symptoms of the condition. It is associated with various syndromes, such as Ehlers–Danlos syndrome, Goldblatt syndrome, Schimke immuno-osseous dysplasia, and Brachio-Skeleto-Genital syndrome [48,52,53].

Dentinal Dysplasia

Dentinal dysplasia (DD) is caused by mutations in genes that are involved in the development of dentin, specifically in odontogenesis [54]. While DD-1 is a genetically heterogenous disease, DD-2 appears to result from dentine sialophosphoprotein (DSPP) mutations [48,55]. There are two types of dentinal dysplasia, type I and type II, and the specific symptoms and severity of the condition can vary widely depending on the type. In type I (radicular DD) dentinal dysplasia, affected teeth have a characteristic “shell-like” and “rootless tooth” appearance due to the lack of root development [9]. In type II dentinal dysplasia, affected teeth have a similar appearance to DI type II, where the primary dentition is more severely affected. Characteristic features involve amorphous, tubule-less dentin, pulpal obliteration, and the opalescent appearance of the tooth [9].

Hypophosphatasia

Hypophosphatasia (HPP) is an uncommon metabolic disorder induced by a mutation in the alkaline phosphatase (ALPL) gene affecting the liver, bones, and/or kidneys [9,56]. Dental features associated with HPP are acellular cementum aplasia or hypoplasia, enlarged pulp chambers linked with faulty dentin mineralization, and early exfoliation of both primary and secondary teeth [9].

#### 3.1.6. Supernumerary Teeth

Supernumerary teeth are extra teeth that develop in the dental arch beyond the normal set of teeth. Supernumerary teeth can be impacted or can erupt into any part of the dental arch, but they are most commonly found in the anterior maxillary region (Figure 2D) [28]. A mesiodens is a type of supernumerary tooth that is located in the midline between the two maxillary central incisors and is the most common type of supernumerary tooth, and it can occur in both primary and permanent dentitions. A mesiodens can cause problems, such as the delayed eruption of permanent incisors, the crowding of teeth, diastema (gaps between teeth), and the misalignment of teeth [57]. In rare cases, a mesiodens can also lead to cyst formation and infection. A paramolar is a supernumerary tooth that forms towards the buccal or palatal of maxillary molars. The most common issue caused by paramolars is the crowding of existing teeth, which can lead to misalignment and improper bite. Paramolars can also cause pain and discomfort if they impinge upon adjacent teeth or if they cause gingival inflammation [58]. In many cases, supernumerary teeth have been reported in patients with different types of disorders and syndromes, including cleidocranial dysplasia [28,57,59,60], Ehlers–Danlos syndrome type III [28,59,60], Ellis–Van Creveld syndrome [28,59,60], Gardner’s syndrome [28,57,59,60], orofaciodigital syndrome type I [28,60], and cleft lip and/or palate [28,57,60]. Supernumerary teeth are associated with mutation in the genes *RUNX2* and *APC* [28,61].

#### 3.1.7. Abnormalities of Jaw Size and Structure

Micrognathia

Micrognathia is a congenital medical condition characterized by an abnormally small mandible or maxilla (Figure 2E). Micrognathia can be caused by various factors, including genetic abnormalities, or problems with the growth and development of the jaw bones during fetal development [62]. It can also occur due to environmental factors, such as exposure to certain medications or toxins during pregnancy [62]. Mutations in more than fifteen different groups of genes have been associated with the development of micrognathia [62]. Micrognathia can occur in isolation or as part of a symptom of other craniofacial conditions. For example, micrognathia is often seen as part of the Pierre Robin sequence that is mostly caused by mutations in *SOX9* [62,63]. The Pierre Robin sequence occurs in about 1 per 8500 live births [64]. It is called a sequence due to a sequence of events that occur during fetal development—the mandible does not grow enough, which causes the tongue to be pushed back, preventing the secondary palatal shelves from developing, leading to a failure of the palatal bones to close and remain separated in the midline. Babies born with the Pierre Robin sequence may have difficulty breathing, feeding, and/or sleeping. Symptoms can range from being very mild to quite severe, including an underdeveloped mandible, cleft palate, glossoptosis, and airway obstruction [64].

Macrognathia

Macrognathia is a rare condition characterized by an abnormally large mandible or maxilla and can be congenital or acquired. Congenital macrognathia is usually caused by genetic abnormalities or developmental disorders, whereas acquired macrognathia may result from conditions such as acromegaly, trauma, or tumor growth [65]. A classic example of macrognathia is seen in the Hapsburg royal family, who exhibited a severe underbite (class III malocclusion) that was inherited over multiple generations in an autosomal-dominant fashion [65].

#### 3.1.8. Bone Disorders

Paget’s Disease of Bone

Paget’s disease (PD), also known as osteitis deformans, is a rare, chronic bone disorder that affects the normal formation and breakdown of bone tissue. It is characterized by the abnormal growth of bone tissue, which can cause bones to become enlarged, weak, and deformed [66,67]. The exact cause of Paget’s disease is unknown, but it is thought to be related to a combination of genetic and environmental factors. Symptoms of Paget’s disease can vary widely but may include bone pain, joint pain and stiffness, bone deformities, hearing loss, and headaches. In some cases, the initial stage of the condition may be asymptomatic and may only be discovered through routine medical imaging [66]. Treatment for Paget’s disease typically involves medications to control bone turnover and pain. Bisphosphonates [67], calcitonin, and other drugs that inhibit bone resorption are commonly used to manage the symptoms, which increase the risk for osteonecrosis of the jaw. In some cases, surgery may be necessary to correct bone deformities or replace joints that have been damaged by the disease. Without treatment, Paget’s disease can lead to complications such as fractures, arthritis, and nerve compression [66]. The occurrence of PD has a familial tendency, and it is linked to polymorphisms in DNA coding in the centrosome structure. As a result, genetics may play a significant part in deciding the prevalence of Paget’s disease [66]. Mutations in the *SQSTM* gene have been strongly associated with the occurrence of Paget’s disease [67,68].

Fibrous Dysplasia

Fibrous dysplasia is a form of benign fibro-osseous disorder characterized by altered osteogenesis. It can be categorized into monostotic (affects a single bone) or polyostotic (affects multiple bones) [67]. The maxilla is more commonly affected when compared to the mandible [67,69]. The disorder is manifested as a painless bone enlargement. The monostotic variant can occur in the jaw, as well as in the ethmoidal or calvarial bones. The polyostotic variants frequently constitute McCune–Albright syndrome (MAS) [67,70]. MAS is a classic triad of café-au-lait skin pigmentation, polyostotic fibrous dysplasia, and peripheral precocious puberty [71]. Most of the features of MAS can be attributed to mutations in *GNAS* [67]. Several mutations can lead to gain of function by causing overactivity in the target tissues, as well as a variety of clinical symptoms that vary in magnitude and age of onset [72].

Cherubism

Cherubism is an extremely rare bone disorder where bone is resorbed only in the jaw bones (mandible and maxilla). The resulting cavities in bone are always symmetrical and fill up with expansive fibro-osseous tissues, giving patients a cherub-like appearance. Cherubism is caused by mutations in the *SH3BP2* gene, which plays a role in regulating bone growth and remodeling [73,74,75]. Symptoms of cherubism usually appear during childhood and may include facial swelling, pain, deformities, and dental problems, such as delayed tooth eruption or displacement of teeth. In some cases, cherubism can also affect the eyes, leading to vision problems or eye movement abnormalities [73,76].

Osteogenesis Imperfecta

Osteogenesis imperfecta (OI) is a rare heritable bone disease that affects 8 in 100,000 people [77]. It is also known as “brittle bone disease”. A dominant mutation in the genes *COL1A1* and *COL1A2* has been associated with OI [77,78,79,80]. Aside from bone fragility, the conventional description of OI manifestation includes blue or grey scleral discoloration and tooth structural defects known as dentinogenesis imperfecta (DI) [78]. OI can be broadly classified into four types, where type 1 is mild, type 2 is neonatal lethal, type 3 is severe, and type 4 is moderately severe [78]. Characteristic features of OI include a reduction in bone mass, skeletal deformities, growth defects, and fragility fractures.

#### 3.1.9. Tongue Anomalies

Ankyloglossia

Ankyloglossia, also known as tongue-tie, is a condition in which the tongue is tethered to the floor of the mouth by a short and tight lingual frenulum, restricting tongue movements [81]. Possible complications associated with ankyloglossia are difficulty in breastfeeding, speech impairment from breastfeeding, speech impairment, or social or mechanical issues, such as the inability to lick the lips or play wind instruments [82]. The milder forms of ankyloglossia might resolve with age, or affected people may learn to adapt effectively to their restricted lingual mobility, while in more impactful cases, frenotomy, frenectomy, or frenuloplasty can be beneficial [83,84,85].

Aglossia

Aglossia is a rare congenital condition denoting an absence of the tongue or a severely underdeveloped tongue. This can result in significant difficulties with speaking, eating, and swallowing. Aglossia may occur on its own or as part of a syndromic condition, such as aglossia–adactylia syndrome or oromandibular limb hypogenesis syndrome [86,87,88].

Bifid Tongue

Bifid tongue, also known as cleft tongue or forked tongue, is a rare condition in which the tongue is split into two distinct sections, giving it a “forked” appearance. Bifid tongue occurs during fetal development when the tongue fails to fuse properly at the lingual septum. In most cases, bifid tongue does not cause any significant health problems, and patients with this condition can eat, speak, and swallow normally. However, in some cases, bifid tongue may be associated with cleft palate or other genetic disorders [89].

Macroglossia

Macroglossia is a condition in which the tongue is abnormally large in proportion to the oral cavity and may cause functional and/or esthetic concerns. It can be congenital or acquired. In the pediatric population, macroglossia can be a sign of an underlying condition, such as Down syndrome [90,91], Beckwith–Wiedemann syndrome (BWS) [90,92], or mucopolysaccharidosis (Maroteaux–Lamy syndrome) [90,93]. In adults, macroglossia can be a symptom of conditions such as hypothyroidism, amyloidosis, or acromegaly [90,94].

Fissured Tongue

Fissured tongue, also known as lingua plicata, is a benign condition in which the surface of the tongue appears to be grooved or furrowed. The fissures or grooves can vary in depth and can run parallel or perpendicular to each other. Fissured tongue is a relatively common condition with an incidence of 5–10% in the global population [95]. It is usually not painful or associated with any significant symptoms, but there may be some discomfort or sensitivity when eating certain foods. Fissured tongue is often seen in families and is more common in people with certain conditions, such as Down syndrome [96,97,98], Melkersson–Rosenthal syndrome [96,99], or psoriasis [96,100,101].

#### 3.1.10. Odontogenic Tumors and Cysts

Odontogenic tumors are a heterogeneous group of tumors that originate from the tissues that form teeth and other structures related to teeth, such as the jawbone and oral mucosa [102]. These tumors can be benign or malignant and can occur at any age, although they are most commonly seen in adults [103]. Although the etiology of most odontogenic tumors is unknown, there have been significant studies identifying the genetic underpinnings of particular odontogenic tumors [104]. Some common types of odontogenic lesions are as follows:

Ameloblastoma

This is a slow-growing, locally invasive, benign tumor that arises from the cells that form the enamel of the teeth. It has a global incidence rate of 0.92 cases per million person-years [105]. Ameloblastoma constitutes approximately 10% of the odontogenic tumors [105]. It is most commonly found in the mandibular molar region [106]. The exact etiology of ameloblastoma is unknown, but mutations in genes involved in the MAPK pathway, such as *BRAFV600E*, are associated with ameloblastomas [105,107,108]. It usually causes a painless swelling resulting in facial asymmetry, malocclusion, or the loosening of teeth;

Odontoma

These are the most common odontogenic tumors, and the exact cause is unknown [109,110]. They are benign tumors composed of dental tissues such as enamel, dentin, the cementum, and pulp. They are classified into two types: complex odontomas and compound odontomas [111]. Odontomas are generally evident radiographically as multiple unerupted teeth. It can cause tooth displacement and malocclusion [112]. Odontomas are often associated with Gardner’s syndrome and Herman’s syndrome [109,113]. A few cases have also been seen in association with Rubinstein–Taybi syndrome [114,115];

Cementoma

This is a benign tumor that arises from the cementoblasts that form the cementum. The cementum is anatomically and functionally connected to the periodontal ligament, which helps anchor the root of the tooth to the adjacent alveolar bone. Cementomas can cause pain and swelling [116]. A probable relation between mutations in *ANO5* and familial gigantiform cementoma has been reported by a few studies [117,118,119];

Dentigerous Cyst

This is one of the most common odontogenic developmental cysts that originates from remnants of the reduced enamel epithelium [120]. It is a fluid-filled sac that develops around an unerupted tooth (Figure 2F,G). It is usually benign but can cause damage to surrounding teeth and bones [121]. Mutations in the gene *PTCH* and polymorphism in chromosome 1qh+ have been found in patients with dentigerous cysts [121,122];

Ameloblastic Fibroma

This is a benign tumor that arises from the cells that form the enamel and dentin of teeth. Ameloblastic fibromas are uncommon, accounting for approximately 2% of all odontogenic tumors [123,124]. The tumors have been designated as childhood and adolescent tumors because they occur virtually exclusively in the first and second decades of life [124]. It is usually represented as a painless swelling, more commonly seen in the mandible compared to the maxilla (Figure 2H) [125];

Odontogenic Myxoma

**Figure 3:**
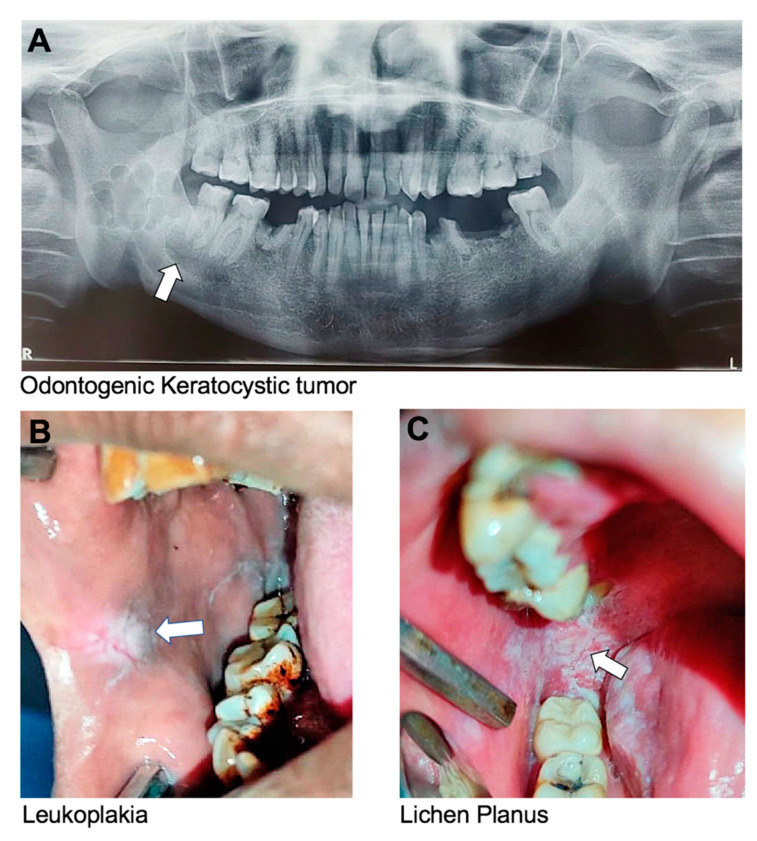
Oral conditions driven mostly by environmental factors with weak genetic contribution, except OKT. (**A**) Odontogenic keratocystic tumor (OKT), (**B**) leukoplakia appears as thick white patches, and (**C**) lichen planus appears as white, itchy bumps of oral mucosa.

This is a benign tumor that arises from the connective tissue that surrounds the teeth. It is locally invasive and has a high recurrence rate [126]. It is rare and can cause bone destruction [127]. Although myxomas are observed in conjunction with mutations in *PRKAR1*, only a few cases of odontogenic myxomas have showed this mutation, and additional research is required to investigate the relationship [128,129];

Keratocystic Odontogenic Tumor (KCOT): 

Previously known as an odontogenic keratocyst, a KCOT is a benign but locally aggressive cystic tumor that originates from the epithelial cells of the dental lamina. It most commonly affects the mandible and can cause bone destruction if left untreated (Figure 3A). In some cases, genetic testing may be recommended for individuals with multiple or recurrent KCOTs, as there is an association with a genetic disorder called nevoid basal cell carcinoma syndrome [106]. A few studies have associated mutations in *PTCH* with KCOT [122,130,131].

#### 3.1.11. Premalignant Conditions and Diseases

Leukoplakia

Leukoplakia is a potentially malignant lesion characterized by white or grayish patches having a mud-crack-like appearance that cannot be scraped off. It forms on the mucous membranes of the mouth, including the tongue, buccal mucosa, and gingiva. The patches are typically thick, raised, and have a rough or scaly texture (Figure 3B) [132]. Leukoplakia is usually painless but may be sensitive to touch or hot, spicy foods. The exact cause of leukoplakia is unknown, but it is believed to be associated with irritants such as tobacco use, alcohol use, and certain viral infections. Long-term use of tobacco is the most common cause of leukoplakia, particularly in the form of chewing tobacco or snuff. Heavy alcohol consumption may also increase the risk of developing leukoplakia, especially when combined with tobacco use. Despite being a possibly malignant disease, the total malignant development of oral leukoplakia is on the order of 5% or higher [133].

Oral Submucous Fibrosis

Oral submucous fibrosis (OSF) is a chronic, progressive disease that affects the oral mucosa. It is characterized by the formation of fibrous bands of tissue in the oral mucosa, which can lead to restricted mouth opening and difficulty eating and speaking. The exact cause of OSF is not fully understood, but it is believed to be associated with the use of betel quid, a substance commonly used in South Asian countries for chewing [134]. Betel quid contains several substances, including areca nut, which is believed to be responsible for fibrosis. Symptoms of OSF may include a burning sensation in the mouth, dryness and a feeling of tightness in the mouth, difficulty opening the mouth, and changes in the texture and color of the oral mucosa. In advanced cases, the fibrous bands may extend into the throat and cause difficulty swallowing [135].

Oral Lichen Planus

Oral lichen planus is a chronic inflammatory condition that affects the oral mucous membrane. Lichen planus can also affect the skin, nails, and hair. Oral lichen planus is characterized by the appearance of white, lacy patches on the labial and buccal mucosa, gingiva, and tongue (Figure 3C) [136,137]. These patches may be painful or cause a burning sensation, particularly when eating or drinking acidic or spicy foods. In more severe cases, oral lichen planus may cause blistering, ulceration, or thickening of the oral mucosa. Oral lichen planus is considered to be an autoimmune condition. Certain medications, such as nonsteroidal anti-inflammatory drugs (NSAIDs), may also contribute to the development of oral lichen planus [138].

#### 3.1.12. Oral and Pharyngeal Cancers

Environmental and behavioral factors, such as tobacco use and infections with the human papillomavirus (HPV), have been implicated in various oral and pharyngeal cancers [139]. Tobacco use is another major cause of several types of cancer, including those affecting the oral cavity, lungs, pharynx, esophagus, bladder, pancreas, and kidneys, among others. Tobacco contains harmful chemicals that can damage DNA and cause mutations, leading to cancer [140]. The oropharyngeal cancers, collectively referred to as head and neck cancer (HNC), are challenging to manage, especially salivary gland cancers. Major salivary gland cancers comprise almost 11% of the total head and neck cancers in the United States [141]. Oral squamous cell carcinoma (OSCC) makes up the majority of HNCs. The survival rates of patients with OSCC have not increased over the past few decades despite improvements in surgery, radiation, and chemotherapy. As a result, a whole new strategy for its treatment that makes use of genetic tools has emerged [142,143].

### 3.2. Genetic Testing for Oral Health Conditions

**Figure 4:**
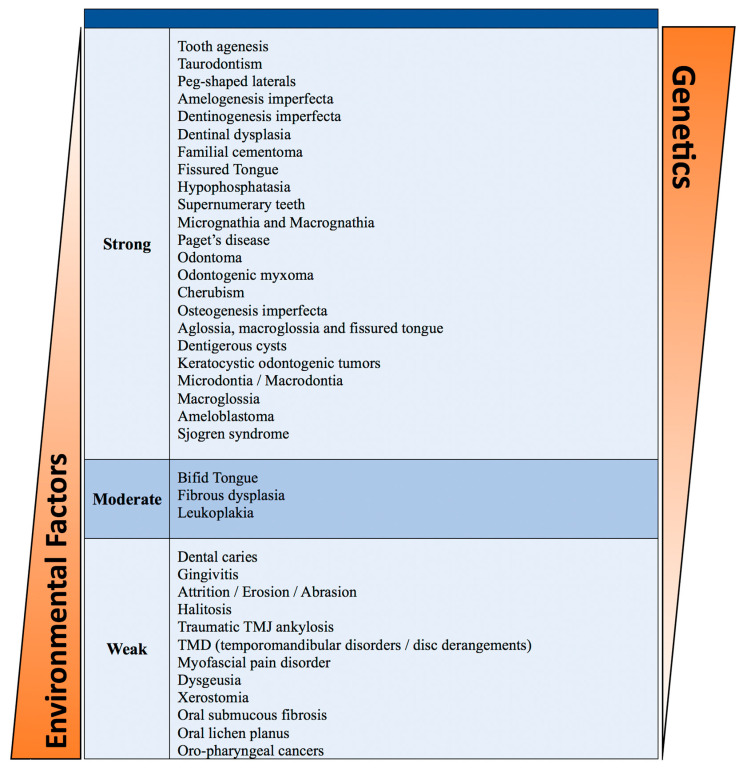
Contribution of genetics and environmental factors to the development and causes of a large list of oral conditions according to the data analysis and evidence strength of the included articles.

Genetic markers can be used to identify individuals who are more likely to experience congenital birth defects or juvenile oral health issues. The American Dental Association (ADA) does not recommend using genetic testing for prognosis and treatment planning. Nevertheless, the ADA does affirm that genetic testing holds potential for clinical application and future treatment planning. Moreover, there are a few dental clinics in the United States that offer genetic testing for aggressive periodontitis (AP) and Sjogren’s syndrome by testing mutations in Cathepsin C *(CTSC)*, *LYST*, *COL5A1*, *FcyIIB*, and *FPRI* for AP. Genetic risk factors have been newly identified for Sjögren’s syndrome, including *IRF5*, *STAT4*, *CXCR5*, *IL12A*, *HLA-DRA*, and *BLK*, which are linked to autoimmune disorders as well [144,145]. In the future, genetic testing for screening and counseling might be recommended to differentiate between oral diseases and conditions that have a strong genetic component and are inherited in a family in a dominant or recessive fashion (Figure 4). This information could be crucial to identify individuals who are at high risk for developing severe oral diseases and disorders with strong genetic components, and for developing preventative and precision dental care to achieve the optimal treatment outcome [146]. An encouraging advancement for genetically complex diseases is the development of the Polygenic Risk Score (PRS), which has emerged as a powerful tool that can assess an individual’s heritable risk of developing a disorder depending on the total number of genetic variants identified in a panel of genes involved in a particular condition [147].

## 4. Conclusions

This systematic review highlights the pivotal role of genetics in shaping oral health outcomes. Through an in-depth analysis of various oral conditions influenced by genetic factors, our study highlights the necessity of integrating genetic considerations into oral healthcare practices. Our findings confirm that an individual’s susceptibility to oral health conditions is significantly influenced by their genetic makeup (Figure 4). These results highlight the need to increase awareness of genetic risk factors for oral diseases and to provide adequate resources for patients, parents of pediatric patients, healthcare providers, and dental hygienists. Early detection of potential conditions through genetic education, counseling, and testing could be vital for enhancing oral healthcare delivery. Nonetheless, the feasibility and ethical implications of genetic testing may pose challenges to implementing these advanced approaches for prevention and early detection.

This review emphasizes the potential utility of genetic testing as a routine diagnostic tool for screening and counseling purposes. As we move towards better oral and overall health for our patients, integrating genetic information into healthcare delivery can pave the way for personalized preventive and treatment plans. While genetics plays a significant role, maintaining good oral hygiene habits, regular dental visits, and healthy lifestyle choices remain essential to minimize the risk of developing oral health problems. The future of oral healthcare should consider both genetic and environmental influences to identify individuals at high risk and develop appropriate preventative and timely treatment plans, ultimately promoting better oral health and overall well-being for our patients.

It is evident that an individual’s oral health may be significantly influenced by their genetic makeup. This evidence makes it important to raise awareness and provide adequate resources to patients, or in the case of pediatric patients, their parents, as well as their healthcare providers, and it aids in the early detection of a potential condition. The roles of genetic education, knowledge, experience, and counseling are therefore critical in oral healthcare delivery. Genetic testing for screening and counseling should emerge as a routine diagnostic aid as we progress on the path toward better oral health and better overall health for our patient populations.

Maintaining good oral hygiene habits, visiting a healthcare provider regularly, and making healthy lifestyle choices can help reduce the risk of developing some oral and systemic health conditions. The future of oral healthcare delivery should consider the genetic component and contribution to identifying individuals at high risk for detrimental oral health disorders to develop appropriate preventive and timely treatment plans.

## Figures and Tables

**Table 1:** A list of retrieved and selected articles in this systematic review study using the NCBI and Cochrane databases for the genetic contribution to a large group of oral conditions.

	NCBI Database			Cochrane Database	
Oral Condition	Retrieved	Included	Evidence Strength (0–5)	Retrieved	Included
Aglossia	17	16	1	0	0
Ameloblastoma	62	53	5	0	0
Amelogenesis imperfecta	103	92	5	2	0
Bifid tongue	12	7	0	0	0
Cherubism	39	34	3	0	0
Dental abrasion	1	0	0	3	0
Dental attrition	10	1	0	0	0
Dental caries	35	4	0	41	0
Dental erosion	1	0	0	3	0
Dentigerous cyst	56	27	2	0	0
Dentinal dysplasia	25	24	2	0	0
Dentinogenesis imperfecta	70	64	5	0	0
Dysgeusia	3	0	0	0	0
Familial cementoma	9	7	0	0	0
Fissured tongue	19	14	1	0	0
Fibrous dysplasia	18	8	0	4	0
Gingivitis	23	4	0	91	1
Halitosis	6	0	0	2	0
Hypophosphatasia	97	84	5	0	0
Impacted teeth	90	22	2	29	0
Keratocystic odontogenic tumor	41	28	2	0	0
Leukoplakia	10	6	0	12	0
Macrodontia	41	32	3	0	0
Macroglossia	41	30	3	0	0
Microdontia	69	62	5	0	0
Micrognathia and macrognathia	15	13	1	1	0
Myofascial pain disorder	6	1	0	15	0
Odontogenic myxoma	15	10	1	0	0
Odontoma	42	32	3	0	0
Oral lichen planus	1	0	0	5	0
Oral submucous fibrosis	1	1	0	2	0
Oropharyngeal cancer	1	1	0	52	0
Osteogenesis imperfecta	18	16	1	20	0
Paget’s disease	24	23	2	15	3
Peg-shaped lateral tooth	11	7	0	0	0
Sjogren syndrome	19	15	1	36	3
Supernumerary teeth	46	35	3	0	0
Taurodontism	65	49	4	0	0
Temporo-mandibular disorders	6	4	0	0	0
Tooth agenesis	47	33	3	0	0
Traumatic TMJ ankylosis	4	0	0	0	0
Xerostomia	16	1	0	77	0
**Total**	**1235**	**860**		**410**	**7**
